# SHH Signaling as a Key Player in Endometrial Cancer: Unveiling the Correlation with Good Prognosis, Low Proliferation, and Anti-Tumor Immune Milieu

**DOI:** 10.3390/ijms251910443

**Published:** 2024-09-27

**Authors:** V. P. Snijesh, Shivakumar Krishnamurthy, Vipul Bhardwaj, K. M. Punya, Ashitha S. Niranjana Murthy, Mahmoud Almutadares, Wisam Tahir Habhab, Khalidah Khalid Nasser, Babajan Banaganapalli, Noor Ahmad Shaik, Walaa F. Albaqami

**Affiliations:** 1 Division of Molecular Medicine, St. John’s Research Institute, St. John’s National Academy of Health Sciences, Bangalore 560034, Karnataka, India; shivakumarsjri@gmail.com; 2 Tsinghua Berkeley Shenzhen Institute, Tsinghua Shenzhen International Graduate School, Tsinghua University, Shenzhen 518055, China; bhardwajvipul27@yahoo.in; 3 Electronics & Communication Engineering, Excel College of Technology, Namakkal 637303, Tamilnadu, India; punyaashokkm@gmail.com; 4 Department of Psychiatry, National Institute of Mental Health And Neuro Sciences, Bangalore 560029, Karnataka, India; ashita.snm@gmail.com; 5 Department of Genetic Medicine, Faculty of Medicine, King Abdulaziz University, Jeddah 21589, Saudi Arabia; malmutadares@kau.edu.sa (M.A.); whabhab@kau.edu.sa (W.T.H.); bbabajan@kau.edu.sa (B.B.); nshaik@kau.edu.sa (N.A.S.); 6 Princess Al-Jawhara Al-Brahim Center of Excellence in Research of Hereditary Disorders, King Abdulaziz University, Jeddah 21589, Saudi Arabia; kknasser@kau.edu.sa; 7 Department of Medical Laboratory Sciences, Faculty of Applied Medical Sciences, King Abdulaziz University, Jeddah 21589, Saudi Arabia; 8 Department of Science, Prince Sultan Military College of Health Sciences, Dhahran 31932, Saudi Arabia

**Keywords:** endometrial cancer, sonic hedgehog pathway, prognostic biomarker, tumor microenvironment, immune enrichment

## Abstract

Endometrial Cancer (EC) is one of the most common gynecological malignancies. Despite its prevalence, molecular pathways, such as the Sonic Hedgehog (SHH) pathway, have not been extensively studied in the context of EC. This study aims to explore the clinical implications of SHH expression in EC, potentially uncovering new insights into the disease’s pathogenesis and offering valuable insights for therapeutic strategies in EC. We utilized data from The Cancer Genome Atlas (TCGA) to divide the dataset into ‘*High SHH*’ and ‘*Low SHH*’ groups based on a gene signature score derived from SHH pathway-related genes. We explored the clinical and tumor characteristics of these groups, focusing on key cancer hallmarks, including stemness, proliferation, cytolytic activity, tumor micro-environment, and genomic instability. ‘*High SHH*’ tumors emerged as a distinct category with favorable clinical and molecular features. These tumors exhibited lower proliferation rates, reduced angiogenesis, and diminished genomic instability, indicating a controlled and less aggressive tumor growth pattern. Moreover, ‘*High SHH*’ tumors displayed lower stemness, highlighting a less invasive phenotype. The immune micro-environment in ‘*High SHH*’ tumors was enriched with immune cell types, such as macrophage M0, monocytes, B cells, CD8 T cells, CD4 T cells, follicular helper T cells, and natural killer cells. This immune enrichment, coupled with higher cytolytic activity, suggested an improved anti-tumor immune response. Our study sheds light on the clinical significance of Sonic signaling in EC. ‘*High SHH*’ tumors exhibit a unique molecular and clinical profile associated with favorable cancer hallmarks, lower grades, and better survival. These findings underscore the potential utility of SHH expression as a robust prognostic biomarker, offering valuable insights for tailored therapeutic strategies in EC. Understanding the SHH pathway’s role in EC contributes to our growing knowledge of this cancer and may pave the way for more effective treatment strategies in the future.

## 1. Introduction

Endometrial cancer (EC) is one of the most common malignancies of female genitalia and second, after cervical cancer, worldwide [1]. Rising EC incidence and mortality rate are major concerns for women, particularly in countries experiencing socioeconomic transitions [2]. The majority of EC patients are elderly women (postmenopausal), particularly those who are in their sixth and seventh decades of life [3]. For over three decades, EC has typically been distinguished into type I and type II [4]. These two classifications differ in terms of epidemiology, histology, prognosis, and treatment [5,6]. Type I ECs are more prevalent, accounting for 80% of diagnosed EC cases, offer favourable prognosis with a 5-year overall survival (OS) rate of 81.3%, and have a recurrence risk of less than 20% [6,7]. Type II ECs are majorly diagnosed at a relatively advanced stage, associated with intermediate-to-poor prognosis with a 5-year OS rate of 55%, and have a high recurrence risk. In recent years, the rapid growth of array- and sequencing-based technologies, coupled with significant advancements in bioinformatics, has revolutionized the landscape of medical research [8]. These developments have enabled researchers to explore the intricacies of the human genome and unlock invaluable insights for understanding and treating complex diseases [9,10,11,12,13,14]. Based on array- and sequencing-based technology, EC has been further classified into four distinct molecular-based subgroups: (i) polymerase ε (POLE) ultra-mutated, (ii) microsatellite instability (MSI) hyper-mutated, (iii) copy-number low (CN LOW), and (iv) copy-number high (CN HIGH) [15,16]. Among these subgroups, POLE mutated shows the best prognosis, followed by MSI, copy-number low, and copy-number high, which has the poorest prognosis [17]. The recent molecular and genomic classification of EC has significantly improved its prognosis and led to the approval of new systemic therapies, as well as the development of precision medicine, resulting in therapeutic advancements and continual adjustments in EC patients’ management. Despite these advances, poorer outcomes are still associated with high-grade, metastatic, and recurrent EC.

In the field of scientific research, it is important to note that we have not delved deeply into understanding the Sonic Hedgehog (SHH) pathway in the context of EC. This study aims to fill that knowledge gap by examining the key genes within the SHH pathway and how they might influence the survival, clinical features, and tumor characteristics of people affected by EC. The SHH pathway is an important fundamental conserved morphogenic pathway that is required for all stages of life, including embryonic development and adult tissue maintenance, along with patterning and tissue differentiation [18,19]. In humans, HH pathway generally involves three secreted HH ligands: Sonic Hedgehog (SHH), Indian Hedgehog (IHH), and Desert Hedgehog (DHH), which are highly conserved between organisms and exhibit catalytic capacity [20]. Among all ligands, Shh is the most widely distributed in human tissues and cells, participates in gene transcription, controls the expression of cytokines and functional proteins, and is crucial in regulating several biological events [21]. DHH and IHH have been reported to play key roles in the proper tissue development of the pancreas, testis, and bone [22,23,24,25]. These ligands bind to the 12-transmembrane glycoprotein receptor patched 1 (PTCH1) that triggers the phosphorylation of the 7-transmembrane helix G protein-coupled receptor, smoothened (SMO), which further facilitates its downstream mediator’s glioma-associated oncogene (GLI) family transcription factors [26]. The GLI transcription factors (GLI1, GLI2, and GLI3) translocate into the nucleus, where they promote cell growth, survival, and differentiation by inducing the expression of target genes [27]. After SHH binds to PTCH1, the primary cilium, a membrane-bound, microtubule-based projection functions as the central hub of signal initiation by releasing SMO from PTCH1-mediated signal repression.

Previous studies, such as those by Feng et al. (2007) and Inoue et al. (2016), have highlighted the critical role of SHH signaling in EC [28,29]. These studies examined the expression of key SHH pathway components—Shh, Ptch, Smo, and Gli1—in endometrial tissues and EC cell lines, exploring their influence on cell proliferation. However, these analyses primarily focused on immunohistochemical expression and did not investigate whether the SHH pathway was actively driving tumor progression. Moreover, they did not leverage large-scale datasets, such as TCGA, to explore these findings in broader cohorts. Our study fills this gap by utilizing high-throughput sequencing data to stratify samples into SHH-activated and non-activated groups. This approach reveals distinct differences in tumor behavior based on SHH signaling status, providing a clearer understanding of its role in EC.

Considering the need for emerging reliable prognostic and/or predictive markers in the management of EC, current research is focusing on more pragmatic approaches. In line with this, and given the limited information on the role that Hedgehog may play in EC, we performed an integrated systemic approach to correlate SHH pathways with clinicopathologic characteristics of EC.

## 2. Results

### 2.1. Comprehensive Dataset Overview and Clinical Characteristics

**Figure 1:**
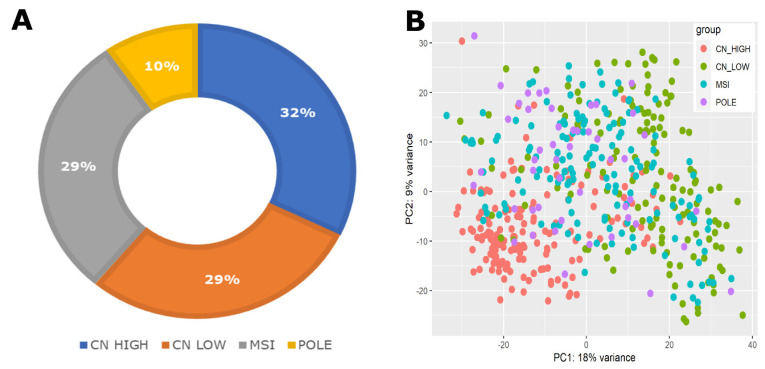

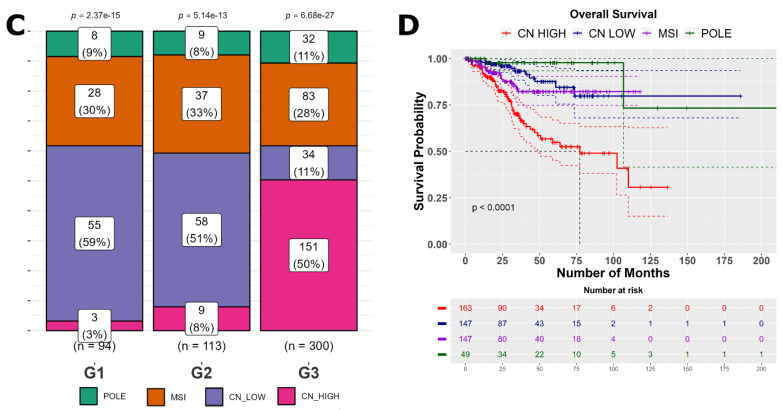
Summary of the EC sample data. (**A**) Distribution of molecular subtypes of EC samples. (**B**) The PCA plot of EC samples based on their molecular subtypes. (**C**) EC tumor grade distribution from low (G1) to high (G3). (**D**) Kaplan–Meier overall survival plot of EC patients showing significantly poor prognosis of the CN HIGH molecular subtype patients.

The gene expression profiles obtained from the GDC portal initially included 589 patient samples, of which only 529 specimens were associated with clinical information. Subsequently, we filtered the datasets based on subtype classification, resulting in a final dataset of 507 samples that included clinical, expression, and subtype information. These filtered samples underwent normalization and variance stabilization using the R package DESeq2 [30,31]. The distribution of EC subtypes within this dataset indicated the following frequencies: CN HIGH (163 samples), CN LOW (147 samples), POLE (49 samples), and MSI (148 samples), represented in Figure 1A. Furthermore, principal component analysis (PCA) has clearly distinguished between various subtypes, with CN HIGH displaying a particularly pronounced separation from CN LOW and the other subtypes, as depicted in Figure 1B. In the CN HIGH subtype, there was a notable predominance of high-grade tumors, while the remaining subtypes exhibited a higher representation of lower-grade tumors (Figure 1C). The survival analysis conducted on these subtypes revealed a noteworthy finding, with the CN HIGH subtype exhibiting the worst prognosis among all subtypes, as evidenced in Figure 1D. These results collectively emphasize the clinical significance of the EC subtypes, with CN HIGH exhibiting particularly adverse survival outcomes.

### 2.2. Correlation Analysis and Composite Gene Signature Score for SHH Pathway Genes

**Figure 2:**
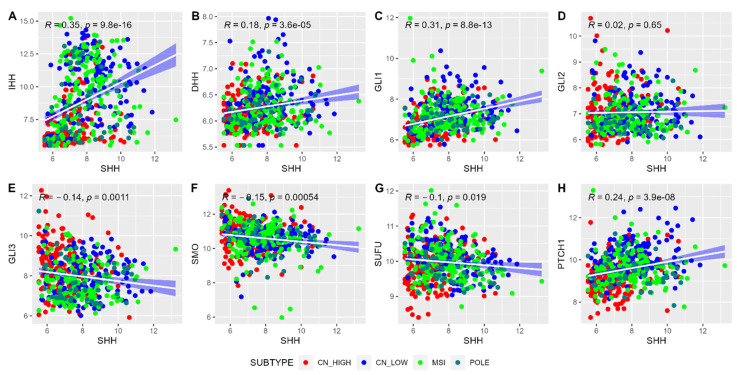
The correlation analysis of genes in the Sonic Hedgehog pathway with transcript expression of *SHH* gene. Each point on the scatterplot represents a specific gene in the SHH pathway, with the *x*-axis denoting the expression levels of these genes, and the *y*-axis indicating the expression level of the *SHH* gene. The plot illustrates the strength and direction of the correlation between *SHH* and the other genes in the pathway. The pattern of points on the plot reveals whether the expression of *SHH* correlates positively, negatively, or not at all with the expression of other genes within the SHH pathway.

Examination of the correlation between the *SHH* gene and other chief genes within the SHH pathway was undertaken, as illustrated in Figure 2. Notably, genes such as *IHH*, *DHH*, *GLI1*, and *PTCH1* displayed highly significant positive correlations with the transcript expression of *SHH*, as evidenced by *p*-values less than 0.001. To gather these relationships into a concise metric, we computed the mean expression levels of these correlating genes, thereby creating a composite gene signature score. The mean expression value of SHH and its correlated genes were extracted from the normalized count, ranging from 6.19 to 10.21. EC tumor samples were further divided into ‘*Low SHH*’ and ‘*High SHH*’ groups based on the 33rd and 66th quantile cut-off of the mean expression value. This categorization method, built into the robust correlations observed, holds promise for determining the influence of the SHH pathway in EC.

### 2.3. Prognostic Implications of High SHH and Low SHH Tumors Levels in Cancer

**Figure 3:**
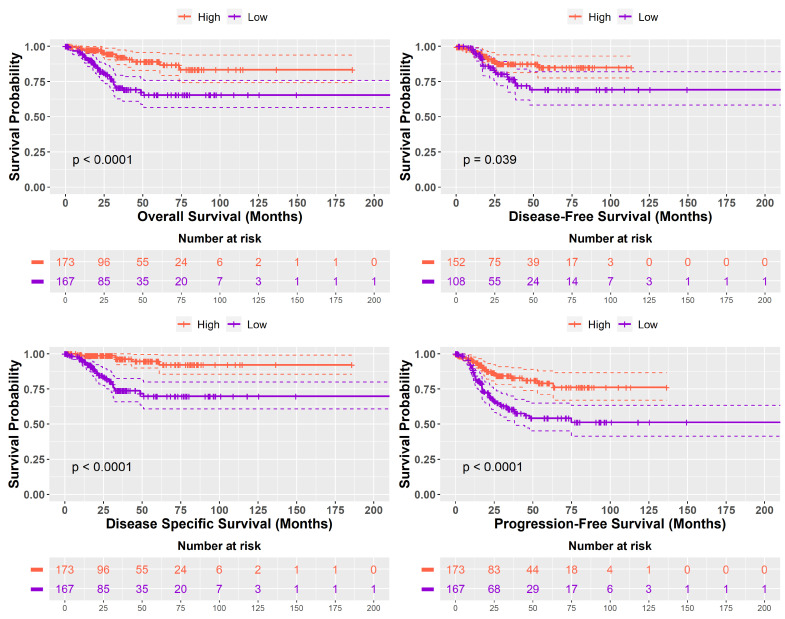
Kaplan–Meier survival curves compare the survival outcomes of two distinct groups: tumor samples with ‘*High SHH*’ group and those with ‘*Low SHH*’ group. The *x*-axis represents time, typically measured in months, and the *y*-axis represents the probability of survival or disease-free status. The two survival curves, one for ‘*High SHH*’ and the other for ‘*Low SHH*’, show how the probability of survival or disease-free status changes over time. These curves visually illustrate the significant differences in survival outcomes between the two groups, as described in the text.

In a comparative survival analysis, tumor samples with ‘*High SHH*’ groups exhibited notably longer prognoses when compared with samples with ‘*Low SHH*’ groups. ‘*High SHH*’ groups were associated with significantly improved outcomes across multiple survival metrics: Overall Survival (*p* < 0.0001), Disease-Free Survival (*p* < 0.039), Disease-Specific Survival (*p* < 0.0001), and Progression-Free Survival (*p* < 0.0001). These findings underscore the clinical significance of SHH signaling levels in predicting favourable patient outcomes and suggest its potential utility as a prognostic biomarker. Kaplan–Meier curves illustrating these differences are depicted in Figure 3.

### 2.4. Clinical Characteristics of SHH High and SHH Low Tumors

**Figure 4:**
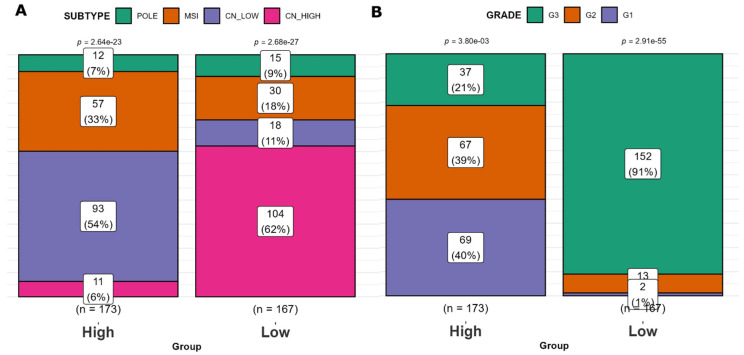
Clinical characteristics of ‘*High SHH*’ and ‘*Low SHH*’ groups. (**A**) Distribution of EC molecular subtypes in ‘*High SHH*’ and ‘*Low SHH*’ groups. The proportions of CN HIGH, CN LOW, POLE, and MSI subtypes are represented in ‘*High SHH*’ and ‘*Low SHH*’ EC tumor groups. (**B**) Tumor grade distribution in ‘*High SHH*’ and ‘*Low SHH*’ groups. The distribution of tumor grades (Grade 1, Grade 2, and Grade 3) is shown for ‘*High SHH*’ and ‘*Low SHH*’ groups.

The distribution of the ‘*High SHH*’ group within the molecular subtypes of EC was as follows: CN HIGH (6%), CN LOW (54%), POLE (7%), and MSI (33%). In contrast, the ‘*Low SHH*’ group of EC tumors exhibited a different distribution among these subtypes: CN HIGH (62%), CN LOW (11%), POLE (9%), and MSI (18%) (Figure 4A). When considering tumor grade, the ‘*High SHH*’ EC tumors showed the following grade distribution: Grade 1 (40%), Grade 2 (39%), and Grade 3 (21%). On the other hand, the ‘*Low SHH*’ EC tumors were distributed as follows: Grade 1 (1%), Grade 2 (6%), and Grade 3 (91%) (Figure 4B). This analysis unveils noteworthy trends. The ‘*Low SHH*’ group consists of a higher proportion of samples from the CN HIGH subtype, which is associated with poor prognosis. In contrast, the ‘*High SHH*’ group is more prevalent in the CN LOW, POLE, and MSI subtypes. Additionally, the ‘*Low SHH*’ group is characterized by a notable predominance of high-grade tumors, while the ‘*High SHH*’ group exhibits a higher representation of lower-grade tumors. These findings underscore the potential clinical relevance of SHH groups in the context of tumor subtypes and grades, which may have implications for prognosis and therapeutic strategies.

### 2.5. Understanding the Clinical Implications of ‘High SHH’ and ‘Low SHH’ Tumors

**Figure 5:**
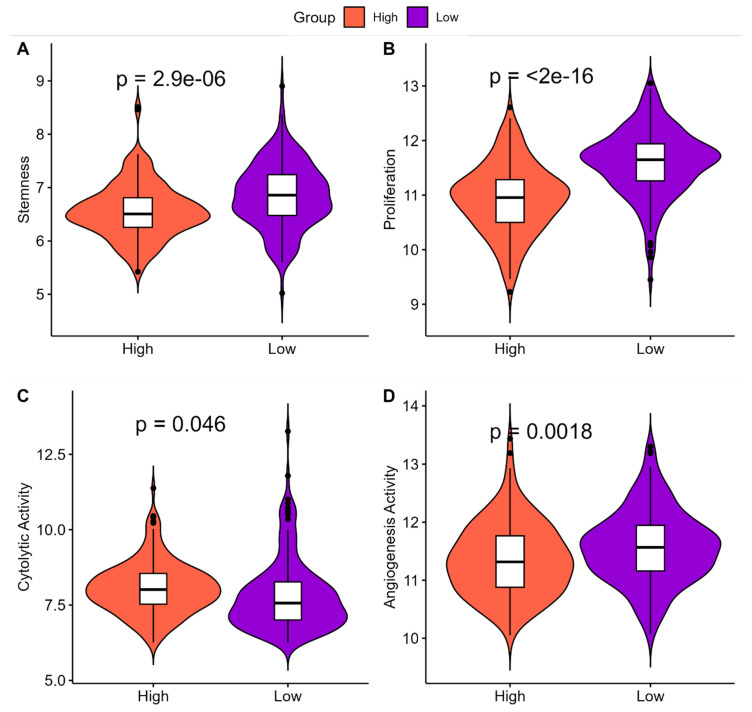
Comparison of tumor characteristics in ‘*High SHH*’ and ‘*Low SHH*’ Tumors. (**A**) This figure illustrates the differences in stemness levels between ‘*High SHH*’ and ‘*Low SHH*’ tumors in EC. ‘*High SHH*’ tumors display lower stemness, suggesting reduced self-renewal and differentiation potential. (**B**) Comparison of Proliferation in ‘*High SHH*’ and ‘*Low SHH*’ Tumors which highlights the variations in proliferation rates between ‘*High SHH*’ and ‘*Low SHH*’ tumors in EC. ‘*High SHH*’ tumors exhibit lower proliferation, indicating a slower growth pattern. (**C**) Comparison of Cytolytic Activity in ‘*High SHH*’ and ‘*Low SHH*’ tumors which showcases the distinctions in cytolytic activity between ‘*High SHH*’ and ‘*Low SHH*’ tumors. ‘*High SHH*’ tumors are associated with higher cytolytic activity, reflecting a stronger anti-tumor immune response. (**D**) Comparison of Angiogenesis Activity in ‘*High SHH*’ and ‘*Low SHH*’ tumors which presents the differences in angiogenesis activity between ‘*High SHH*’ and ‘*Low SHH*’ tumors. ‘*High SHH*’ tumors exhibit lower angiogenesis activity, implying a potential decrease in tumor invasiveness.

The molecular and clinical characteristics associated with ‘*High SHH*’ and ‘*Low SHH*’ tumors in EC revealed several important insights into their clinical impact. ‘*High SHH*’ tumors exhibited significantly lower stemness compared to ‘*Low SHH*’ tumors, indicating a reduced capacity for self-renewal and differentiation (Figure 5A). Additionally, ‘*High SHH*’ tumors were associated with lower proliferation rates, suggesting a reduced rate of cell division and replication relative to ‘*Low SHH’* tumors (Figure 5B). Furthermore, ‘*High SHH*’ tumors displayed higher cytolytic activity, indicating a stronger immune response in comparison to ‘*Low SHH*’ tumors (Figure 5C). Lastly, angiogenesis activity was found to be lower in ‘*High SHH*’ tumors, pointing to reduced blood vessel formation when compared to ‘*Low SHH*’ tumors (Figure 5D). Overall, ‘*High SHH*’ tumors in EC exhibit characteristics that are generally associated with more controlled growth, reduced aggressiveness, enhanced immune response, and less invasive behavior. These features suggest that ‘*High SHH*’ tumors may have a more favorable clinical prognosis compared to ‘*Low SHH*’ tumors.

### 2.6. Genetic Stability and Tumor Mutational Burden in ‘High SHH’ Tumors

**Figure 6:**
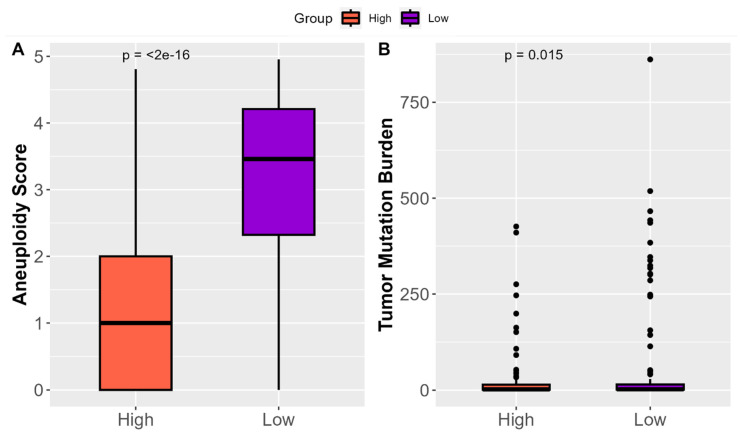
Influence of ‘*High SHH*’ tumors on Genetic Alterations in Tumors. (**A**) The results of aneuploidy assessment, highlighting the significantly reduced aneuploidy scores observed in ‘*High SHH*’ tumors in EC. Reduced aneuploidy reflects a more stable and balanced chromosomal composition, suggesting a controlled tumor growth pattern. (**B**) The outcomes of TMB analysis, indicating that ‘*High SHH*’ tumors display lower TMB. The lower TMB signifies a reduced frequency of genetic mutations within the tumor cells, which may contribute to a less aggressive and slower-growing tumor phenotype.

The examination of ‘*High SHH*’ tumors in the context of genetic alterations within tumors provides valuable insights into their clinical impact. An assessment of aneuploidy, which measures the degree of abnormal chromosome numbers in cancer cells, revealed that ‘*High SHH*’ tumors exhibited significantly reduced aneuploidy scores (Figure 6A). This finding suggests a more stable and balanced chromosomal makeup in ‘*High SHH*’ tumors. Reduced aneuploidy is often associated with a more controlled and less aggressive tumor growth pattern, which may lead to improved clinical outcomes for patients with ‘*High SHH*’ tumors. The analysis of Tumor Mutational Burden (TMB), which measures the frequency of genetic mutations within a tumor, demonstrated that ‘*High SHH*’ tumors displayed lower TMB (Figure 6B). This indicates a lower occurrence of genetic mutations within the tumor cells. Overall, ‘High SHH’ tumors exhibit significantly reduced aneuploidy scores, reflecting a more stable chromosomal makeup and lower Tumor Mutational Burden (TMB), indicating fewer genetic mutations within the tumor cells.

### 2.7. Impact of ‘High SHH’ Tumors on Immune Cell Composition

**Figure 7:**
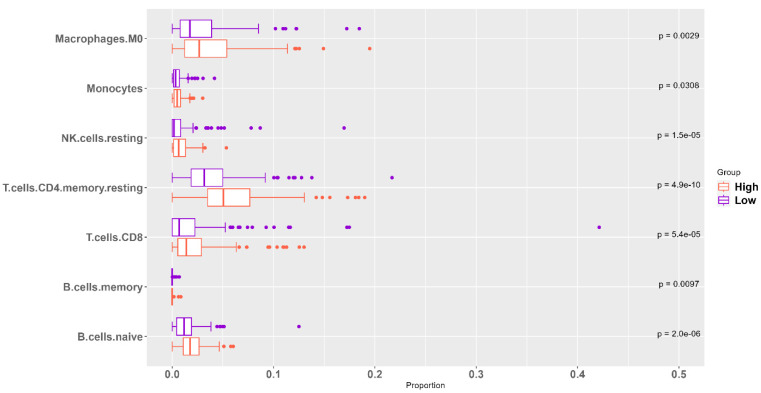
Immunological Profile of ‘*High SHH*’ Tumor’s Microenvironment. ‘*High SHH*’ tumors exhibit elevated levels of various immune cell types, including B cells, CD8+ T cells, CD4+ T cells, follicular helper T cells, natural killer cells, monocytes, and macrophages. The abundance of these immune cell populations suggests a complex and potentially significant immune response within the tumor microenvironment, with potential implications for tumor immunogenicity and therapeutic strategies.

The association between ‘*High SHH*’ tumors and the immune microenvironment holds significant clinical implications. ‘*High SHH*’ tumors are characterized by a distinct immune profile with higher levels of various immune cell populations compared to ‘*Low SHH*’ tumors. This elevated presence of immune cells includes macrophage M0, monocytes, B cells, CD8 T cells, CD4 T cells, follicular helper T cells, and natural killer cells (Figure 7). These findings suggest a more robust and diversified immune response within the tumor microenvironment of ‘*High SHH*’ tumors. The presence of higher numbers of immune cell types, such as CD8 T cells, CD4 T cells, and natural killer cells, can enhance the body’s ability to recognize and destroy cancer cells, potentially leading to improved anti-tumor activity. Macrophage M0 and monocytes are important components of the innate immune system, contributing to the initial response against cancer cells. Additionally, the presence of B cells and follicular helper T cells may indicate a more coordinated and adaptive immune response, which can play a crucial role in regulating and enhancing the body’s immune defense mechanisms against cancer. Based on the findings, ‘*High SHH*’ tumors appear to be associated with a more robust and diversified immune response, which could be interpreted as having a potential anti-tumorigenic effect. The elevated presence of various immune cell populations suggests enhanced immune surveillance and response against cancer cells, potentially contributing to a more controlled tumor growth and improved clinical outcomes.

### 2.8. Differentially Expressed Genes and Pathways

**Figure 8:**
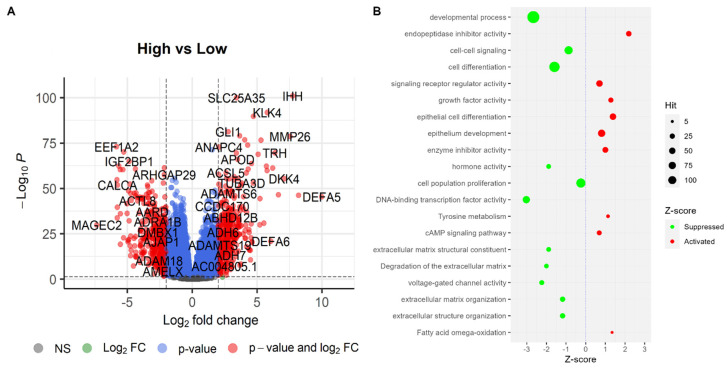
Differentially expressed genes and pathways associated with them. (**A**) Volcano plot representing DEGs. In the plot, genes that are significantly upregulated are on the right side (positive fold change), while downregulated genes are on the left side (negative fold change). (**B**) Represents the outcomes of pathway enrichment analysis, which identifies biological pathways or processes significantly affected by the differentially expressed genes.

Differential gene expression analysis has been influential in identifying key molecular changes associated with ‘*High SHH*’ tumors in endometrial tumors. The study revealed that there were 395 upregulated genes and 423 downregulated genes in ‘*High SHH*’ endometrial tumors, as illustrated in the volcano plot (Figure 8), where a fold change of 2 and an adjusted *p*-value of 0.05 were employed as the criteria for differential expression. The upregulated genes in ‘*High SHH*’ tumors are associated with a diverse array of biological processes and molecular functions. Notably, the cAMP signaling pathway, a pivotal cellular pathway involved in regulating various cellular responses, showed upregulation. This finding suggests that SHH may influence cellular responses through this pathway in EC. Additionally, the upregulation of genes related to epithelium development, enzyme inhibitor activity, tyrosine metabolism, and growth factor activity may indicate a role for SHH in promoting cell growth and differentiation. Furthermore, the upregulation of genes associated with fatty acid omega-oxidation implies potential alterations in cellular energy metabolism within ‘*High SHH*’ endometrial tumors, which could be a significant factor in their pathogenesis.

Conversely, the downregulated genes in ‘*High SHH*’ endometrial tumors reflect a different set of biological processes and molecular functions. Notably, pathways related to DNA-binding transcription factor activity, developmental processes, voltage-gated channel activity, and extracellular matrix degradation were downregulated. These changes might indicate an impact on cellular differentiation, tissue development, and the extracellular microenvironment. Furthermore, the downregulation of genes associated with hormone activity and cell population proliferation in ‘*High SHH*’ tumors suggest potential alterations in hormonal regulation and a decreased propensity for uncontrolled cell growth.

### 2.9. Impact of Variables on Survival Outcomes in Endometrial Cancer

**Figure 9:**
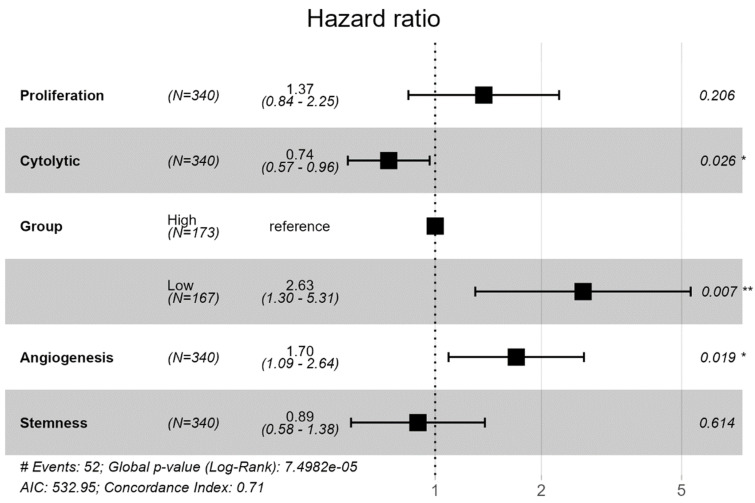
Cox proportional hazards model results showing the impact of various variables on survival outcomes in a cohort of endometrial cancer. (** *p* < 0.01; * *p* < 0.05).

We employed a Cox proportional hazards model to investigate the impact of multiple variables on survival outcomes in the cohort of EC (Figure 9). The results of the Cox proportional hazards regression analysis reveal several significant associations between clinical and molecular characteristics and overall survival in the cohort. Notably, individuals categorized in the ‘*Low SHH*’ had a significantly higher hazard of experiencing the event of interest, with a hazard ratio of 2.63 and a significant *p*-value (*p* = 0.007), indicating its strong association with adverse outcomes. Cytolytic activity emerges as another notable factor, with a hazard ratio of 0.7384 (*p =* 0.026). This suggests that higher cytolytic activity is associated with a decreased risk of adverse outcomes, highlighting its potential role in improving patient survival. Angiogenesis with a hazard ratio of 1.70 (*p* = 0.019) suggests that increased angiogenesis is linked to a higher risk of adverse outcomes. Proliferation and stemness did not show statistically significant associations with survival in this analysis. However, there was a trend suggesting that higher levels of these factors may be associated with adverse outcomes. The model displayed a concordance value of 0.71, indicating a reasonable ability to discriminate between different survival times. Additionally, the likelihood ratio, Wald, and Score (logrank) tests collectively affirmed the statistical significance of the model (*p* < 0.001), signifying that at least one predictor significantly influences survival. These findings provide valuable insights into the factors affecting survival in this cohort and underscore the relevance of the ‘*Low SHH*’ variable as a robust predictor of adverse outcomes.

## 3. Discussion

Endometrial cancer, a prevalent gynecological malignancy, has seen significant advances in the understanding of its molecular underpinnings in recent years. Among the molecular pathways implicated in this cancer, the Sonic Hedgehog pathway remains relatively underexplored in the context of EC. The SHH pathway is well-known for its pivotal role in embryonic development and tissue homeostasis [32]. In cancer, it has been extensively studied in various contexts, including basal cell carcinoma and pancreatic cancer. However, its role in EC has gained less attention by comparison. The SHH pathway’s potential involvement in EC presents an interesting avenue for further research, as understanding its contributions to the disease may uncover new therapeutic opportunities and diagnostic strategies. Given the increasing importance of targeted therapies in EC management, the SHH pathway’s exploration in this cancer type holds promise for enhancing our understanding of its molecular complexity and clinical implications.

Previous studies have highlighted the significance of SHH signaling in EC by examining the expression of key SHH pathway components in endometrial tissues and cell lines [28,29]. However, these studies often relied on limited sample sizes and single-gene analyses, hindering a comprehensive understanding of SHH signaling impact on tumor progression. These studies primarily utilized immunohistochemical analysis and did not explore whether the SHH pathway actively drives tumor progression or utilize large-scale datasets such as TCGA. Similarly, investigations by Kim et al. (2009) and Polychronidou et al. (2018) focused on SHH-related markers through IHC or RT-PCR, but were limited to smaller cohorts and single-gene analyses [33,34]. Our study addresses these gaps by leveraging high-throughput sequencing data to stratify samples into SHH-activated and non-activated groups. This methodology reveals significant differences in tumor behavior based on SHH signaling status and provides a more comprehensive understanding of its role in EC. By utilizing a heterogeneous dataset that encompasses various EC subtypes, and by employing a gene signature score developed from multiple genes, our analysis offers a more robust prognostic prediction compared to previous studies. Gene signature scores capture complex gene expression patterns and provide a better understanding of cancer biology than single-gene analyses [35]. This approach is particularly valuable for characterizing diseases driven by intricate gene networks, where individual gene expression alone may not fully capture the disease phenotype.

The partition of tumor samples into ‘*High SHH*’ and ‘*Low SHH*’ categories reveal a notable clinical impact on EC. ‘*High SHH*’ tumors are associated with better prognosis and more favorable cancer hallmarks. These tumors tend to have a higher proportion of lower-grade EC, which typically indicates a less aggressive and more manageable cancer. Furthermore, ‘*High SHH*’ tumors exhibit lower stemness and reduced proliferation rates compared to ‘*Low SHH*’ tumors. Stemness is a measure of a tumor’s potential for self-renewal and differentiation [36]. The lower stemness in ‘*High SHH*’ tumors suggest a reduced capacity for the formation of new cancer cells, which may be associated with a more controlled tumor growth pattern and potentially better clinical outcomes. ‘*High SHH*’ tumors are linked to lower proliferation rates when compared to ‘*Low SHH*’ tumors. Proliferation measures the rate at which cancer cells divide and replicate. The lower proliferation in ‘*High SHH*’ tumors implies that these tumors may be less aggressive and slower growing, which could be associated with a less invasive clinical course and, consequently, potentially improved patient outcomes.

Moreover, ‘*High SHH*’ tumors exhibit higher cytolytic activity compared ‘*Low SHH*’ tumors. Cytolytic activity reflects the tumor’s ability to elicit an immune response, potentially leading to the destruction of cancer cells [35]. Higher cytolytic activity in ‘*High SHH*’ tumors indicates a more robust anti-tumor immune response, which is generally a positive clinical sign as it may enhance the body’s natural defense against cancer. ‘*High SHH*’ tumors are associated with lower angiogenesis activity when compared to ‘*Low SHH*’ tumors. Angiogenesis is the formation of new blood vessels that supply nutrients to the tumor [37]. Reduced angiogenesis in ‘*High SHH*’ tumors may signify a less aggressive and invasive tumor phenotype. Lower angiogenesis is often seen as a favourable clinical characteristic, as it suggests a tumor may be less likely to spread to other parts of the body.

Additionally, ‘*High SHH*’ tumors exhibit a reduced aneuploidy score and lower Tumor Mutational Burden, which are often associated with a more controlled and less aggressive tumor growth pattern. It may also signify that ‘*High SHH*’ tumors have a reduced likelihood of evolving into more aggressive and treatment-resistant forms. Consequently, the lower TMB in ‘*High SHH*’ tumors may have a favorable clinical impact by potentially improving patient outcomes. These features suggest that ‘*High SHH*’ tumors may have a more favorable clinical prognosis compared to other tumor types with higher genetic alterations.

The enrichment of immune cells, including macrophage M0, monocytes, B cells, CD8 T cells, CD4 T cells, follicular helper T cells, and natural killer cells in ‘*High SHH*’ tumors, underscores a more robust and diversified immune response, potentially enhancing the body’s ability to combat cancer. Finally, multivariate analysis identifies ‘*High SHH*’ tumors as having a significantly lower hazard of experiencing adverse events, while ‘*Low SHH*’ tumors are associated with a significantly higher hazard. These findings collectively emphasize the clinical significance of the SHH pathway in EC and highlight its potential utility as a prognostic biomarker, offering valuable insights for tailored treatment strategies.

## 4. Materials and Methods

### 4.1. Collection of Datasets

The gene expression profiles of EC were acquired from the Genomic Data Commons (GDC) Portal, version 32, accessible at https://portal.gdc.cancer.gov/, (accessed on 1 March 2024) using the R package *TCGAbiolinks* [38]. Simultaneously, structured clinical data were obtained from cBioPortal [39]. The gene expression data were downloaded in raw counts format, and these counts were generated through the STAR pipeline [40]. Sample inclusion criteria encompassed samples that contained pertinent information regarding subtype, demographic, and survival details. To identify key genes associated with the Sonic Hedgehog pathway, we extracted relevant data from the Kyoto Encyclopedia of Genes and Genomes (KEGG) [41]. The extracted genes relevant to SHH signaling included *IHH*, *DHH*, *SHH GLI1*, *GLI2*, *GLI3*, *SMO*, *SUFU*, and *PTCH1*.

### 4.2. Data Processing and Normalization

The raw gene expression counts for EC obtained from the GDC Portal were subjected to a series of processing steps using the R package DESeq2 [30]. The processing pipeline included functions such as *estimateSizeFactors*, *estimateDispersions*, and *nbinomWaldTest*. Following this, the data underwent variance stabilizing transformation using the vst module within DESeq2. This transformation facilitated the generation of normalized and variance-stabilized expression values for the genes involved in the Sonic Hedgehog pathway. Subsequently, these values were used to investigate correlations among these genes, aiming to identify regulatory trends [42]. Genes that exhibited significant positive correlations with the transcript expression of SHH were screened for further analysis. The expression values of these genes, which displayed positive correlations with SHH transcripts, were extracted from the normalized counts. To categorize the samples, they were divided into ‘*Low SHH*’ and ‘*High SHH*’ groups based on a median cut-off determined by the mean expression of the positively correlated genes with *SHH*. This categorization allowed for a more in-depth exploration of the relationship between gene expression and the SHH pathway.

### 4.3. Clinical Characteristics

We conducted a Kaplan–Meier survival analysis using data exclusively sourced from the TCGA database to examine the influence of clinical characteristics on the survival rates of individuals with ‘*High SHH*’ and ‘*Low SHH*’ tumors. Kaplan–Meier survival analysis was chosen to assess the association between SHH tumor levels and patient survival over time, allowing us to investigate the potential impact of these characteristics on the patient’s overall survival event.

### 4.4. Cytolytic Activity

The cytolytic activity (CYT) score was determined as a measure of cancer immunity. This score was calculated based on the mRNA expression levels of two crucial genes, *GZMA* and *PRF1*. A higher CYT score indicated a stronger anti-tumor immune response and was associated with improved survival outcomes [35].

### 4.5. Proliferation and Angiogenesis Score

The proliferation score is a key molecular measure in cancer assessment, reflecting how quickly cancer cells divide. High scores indicate aggressive, fast-growing tumors, while low scores suggest slower growth. It guides treatment decisions, calculated from three markers: *MKI67*, *MCM6*, and *PCNA*, helping tailor therapies for better outcomes [35,42]. Angiogenesis, the process of new blood vessel formation, plays a critical role in tumor growth and metastasis by supplying nutrients and oxygen to cancer cells [43,44]. The angiogenesis score was calculated using markers *PECAM1*, *VEGFA*, *ANG2*, *CD105*, *FGF*, and *PDGF*, which are key regulators of this process in the tumor microenvironment [37].

### 4.6. Stemness Score

Cancer stemness highlights the presence of a distinct subpopulation of cells within tumors, akin to stem cells, capable of self-renewal and differentiation. These cancer stem cells often exhibit resistance to conventional cancer treatments. To assess cancer stemness, a stemness score was calculated using the expression levels of key stem cell markers, like *POU5F1*, *SOX2*, *KLF4*, *MYC*, *NANOG*, and *SALL4* [36]. Understanding these mechanisms is vital for the development of targeted therapies aimed at eliminating these resilient cells and preventing tumor relapse.

### 4.7. Genetic Alterations in Tumors

The genetic alterations in the samples were determined by two parameters: Tumor Mutational Burden (TMB) and aneuploidy. Aneuploidy, the presence of an abnormal number of chromosomes, is a hallmark of cancer that contributes to genomic instability and tumor progression [45]. TMB is the total number of mutations per coding region of a tumor genome. The TMB score was calculated by assessing the number of nonsynonymous mutations in the tumor’s exome, thereby providing insight into the tumor’s genetic instability [46]. The details regarding Aneuploidy and TMB for each sample were collected from TCGA database.

### 4.8. Identification of Immune Cell Composition

To estimate the proportions of different immune cell types within the EC transcriptome profile, we utilized the CIBERSORT algorithm, a tool that employs linear support vector regression (SVR) for feature selection and deconvolution of the cell mixture from the gene expression data [47]. Specifically, gene expression profiles of ‘*High SHH*’ and ‘*Low SHH*’ EC samples were fed into the CIBERSORT algorithm, which transformed the gene expression matrix into an immune cell-matrix. The analysis was conducted with filtering criteria involving 1000 permutations, and a significance threshold was set at *p* ≤ 0.05 for robust results.

### 4.9. Identification of Differentially Expressed Genes

The genes that had zero counts across the samples were removed from the analysis. Later, the differential expression analysis was performed using DESeq2 based on the Wald test scores followed by the Benjamini–Hochberg procedure for removing false positives in the data [30]. The genes that had an absolute fold change (FC) of ≥2 and an adjusted *p*-value of ≤0.05 were screened as differentially expressed genes (DEGs). The heatmap and volcano plots representing DEGs were created using R packages like *pheatmap* and *EnhancedVolcano*, respectively.

### 4.10. Functional Enrichment Analysis

Functional enrichment analysis validates the physiological importance of the genes involved in a biological process and helps to reveal unintended gene activity. The functional enrichment analysis of the DEGs was performed using g:Profiler, a webserver used to interpret the function of gene lists (https://biit.cs.ut.ee/gprofiler/gost, accessed on 23 September 2024) [48]. This server matches a queried gene list to established functional data sources and uncovers gene ontologies as well as pathway terms that are significantly enriched at *q* ≤ 0.05.

### 4.11. Analysing Multiple Variables in EC Survival Analysis

We conducted a Cox proportional hazards model analysis to assess the impact of multiple variables on survival outcomes within the EC samples. We evaluated various predictors, including SHH Groups, Proliferation, Angiogenesis, Cytolytic, and Stemness, and determined their influence on survival, although statistical significance varied across these factors. All the relevant information about tumor and clinical characteristics including all generated scores are given in Supplementary File S1.

## 5. Conclusions

Our study explores the relatively unexplored territory of the Sonic Hedgehog (SHH) pathway in the context of Endometrial Cancer. We have unveiled a spectrum of clinically significant findings that highlight the pivotal role of SHH tumors in EC’s molecular landscape and clinical outcomes. ‘*High SHH*’ tumors emerge as a distinct and promising category, showcasing favorable cancer hallmarks, lower grades, reduced stemness, slower proliferation rates, and higher cytolytic activity. These characteristics collectively suggest a more controlled and less aggressive tumor phenotype, which may translate into improved patient prognoses. Additionally, ‘*High SHH*’ tumors exhibit lower angiogenesis activity, reduced aneuploidy, and lower Tumor Mutational Burden, signifying potential clinical advantages. The immune microenvironment in ‘*High SHH*’ tumors is enriched with various immune cell types, which could bolster the body’s defence mechanisms against cancer. Multivariate analysis further substantiates the prognostic significance of the SHH tumors, where ‘*High SHH*’ tumors demonstrate a significantly lower hazard of adverse events. Our study underscores the potential utility of SHH gene signature as a robust prognostic model and provides a foundation for future investigations and tailored therapeutic strategies in EC.

## Data Availability

The data presented in this study are available in GDC Data Portal and cBioPortal at https://portal.gdc.cancer.gov/ (accessed on 23 September 2024) and https://www.cbioportal.org/ (accessed on 23 September 2024), respectively.

## Supplementary Materials

The supporting information can be downloaded at https://www.mdpi.com/article/10.3390/ijms251910443/s1.
